# Evolution of feeding specialization in Tanganyikan scale-eating cichlids: a molecular phylogenetic approach

**DOI:** 10.1186/1471-2148-7-195

**Published:** 2007-10-18

**Authors:** Rieko Takahashi, Katsutoshi Watanabe, Mutsumi Nishida, Michio Hori

**Affiliations:** 1 Department of Zoology, Division of Biological Science, Graduate school of Science, Kyoto University, Kitashirakawa-Oiwake, Sakyo, Kyoto 606-8502, Japan; 2 Ocean Research Institute, University of Tokyo, 1-15-1 Minamidai, Nakano, Tokyo 164-8639, Japan

## Abstract

**Background:**

Cichlid fishes in Lake Tanganyika exhibit remarkable diversity in their feeding habits. Among them, seven species in the genus *Perissodus *are known for their unique feeding habit of scale eating with specialized feeding morphology and behaviour. Although the origin of the scale-eating habit has long been questioned, its evolutionary process is still unknown. In the present study, we conducted interspecific phylogenetic analyses for all nine known species in the tribe Perissodini (seven *Perissodus *and two *Haplotaxodon *species) using amplified fragment length polymorphism (AFLP) analyses of the nuclear DNA. On the basis of the resultant phylogenetic frameworks, the evolution of their feeding habits was traced using data from analyses of stomach contents, habitat depths, and observations of oral jaw tooth morphology.

**Results:**

AFLP analyses resolved the phylogenetic relationships of the Perissodini, strongly supporting monophyly for each species. The character reconstruction of feeding ecology based on the AFLP tree suggested that scale eating evolved from general carnivorous feeding to highly specialized scale eating. Furthermore, scale eating is suggested to have evolved in deepwater habitats in the lake. Oral jaw tooth shape was also estimated to have diverged in step with specialization for scale eating.

**Conclusion:**

The present evolutionary analyses of feeding ecology and morphology based on the obtained phylogenetic tree demonstrate for the first time the evolutionary process leading from generalised to highly specialized scale eating, with diversification in feeding morphology and behaviour among species.

## Background

Cichlid fishes in the East African Great Lakes exhibit a remarkable diversity of feeding ecology, morphology and behaviour [1]. Lake Tanganyika, the oldest of these lakes with an estimated age of 9–12 Myr [2], contains the the most morphologically and ecologically complex species flock of cichlid fishes [3]. Scale-eating cichlids of the tribe Perissodini is prehaps among the most specialized cichlids [1]. The endemic tribe Perissodini comprises nine species [4,5]. Liem & Stewart [6] classified them into two genera, *Perissodus*, which includes all scale eaters identified so far in the lake, and *Haplotaxodon*. We follow their classification here, although Poll [5] further subdivided the former genus into three genera, *Perissodus *(*P. microlepis *and *P. eccentricus*), *Plecodus *(*P. multidentatus*, *P. paradoxus*, *P. elaviae*, *P. straeleni*) and *Xenochromis *(*P. hecqui*).

The scale eaters are characterised by unique oral jaw teeth that vary in shape among species, showing functional specialization to scale eating [6]. Among the species, *P. microlepis *and *P. straeleni*, which syntopically inhabit shallow rocky regions of the lake [7], show specialized feeding techniques, employing a variety of specialized morphologies such as mimicking body colour morphs of prey species [8], or jaw asymmetries with the jaw opening either to the left or right [9].

Although the origin of such peculiar scale-eating habits has long been questioned, and the ancestors of scale eaters have been variously suggested as being algae eaters, ectoparasite feeders, or piscivorous species [1,10], the answer remains unclear. This is partly because the other five *Perissodus *species are deepwater inhabitants whose ecology is poorly known [11,12]. Above all, a reliable phylogenetic framework, which is essential for evolutionary analyses, has yet to be obtained for the Perissodini.

Previous molecular phylogenetic studies of the Tanganyikan cichlids have placed the tribe Perissodini within the "H-lineage" [13,14]. The phylogenetic relationships within the Perissodini have also been inferred from morphological aspects [6]. However, the small number of shared characters between species and the inclusion of adaptive traits (e.g., oral tooth shape) may hinder the construction of reliable phylogenetic relationships from morphology. A molecular phylogenetic approach using mitochondrial DNA (mtDNA) also has potential problems, sometimes resulting in patterns incongruent with species morphology or nuclear DNA when hybridisation or incomplete lineage sorting is involved [15,16]. A recent mtDNA-based phylogenetic study for Perissodini showed a strong discordance in species relationships from those of morphological and nuclear phylogenetic trees [17]. Mitochondrial DNA and any single locus would be susceptible to this problem [18-20]. In recent years, the amplified fragment length polymorphism (AFLP) method, in which large numbers of restriction fragments from whole genome digests can be examined, has provided powerful phylogenetic markers to overcome the above problems and is especially useful for analyses of closely related cichlid species [21-23]. Koblmüller *et al*. [17] also used this method for Perissodini, although the phylogenetic relationships within *Perissodus *still remain to be clarified since one specialized scale eater, *P. eccentricus*, was missing from their AFLP analysis, and a greater number of AFLP characters might be necessary to resolve the phylogenetic relationships of recently diverged species.

This study aims to clarify the evolution of scale eating in Tanganyikan cichlids, *Perissodus *species. For that purpose, we conducted molecular phylogenetic analyses based on over 1000 multilocus analyses using AFLP data for all known Perissodini species. In addition, we examined stomach contents, oral jaw tooth morphology, and habitat depths. Subsequently, we compared these characteristics based on the obtained phylogenetic framework. Finally, we discuss the evolutionary process of specialization to scale eating and associated feeding morphologies and feeding behaviours.

## Results

### AFLP phylogeny

**Figure 1:**
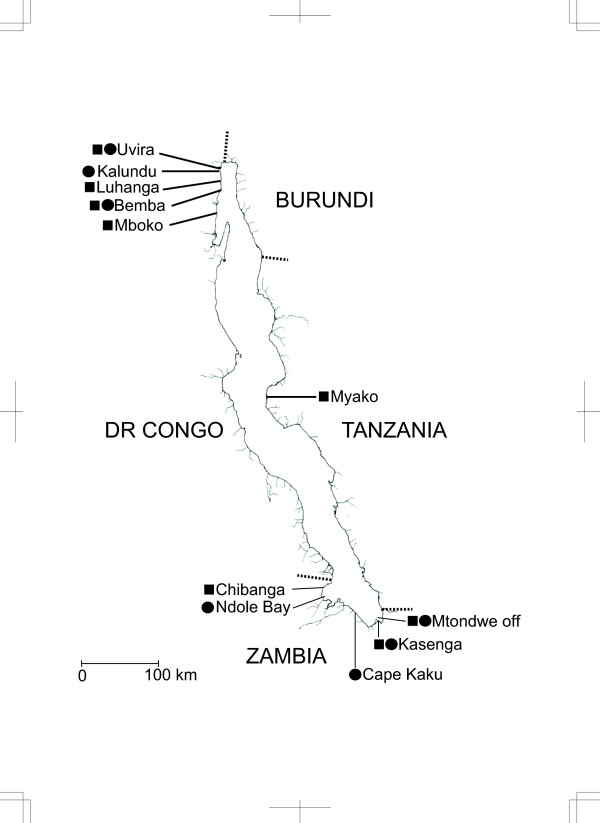
**Map of Lake Tanganyika**. Sampling sites of specimens used in phylogenetic analyses (circles) and stomach contents analyses (squares).

**Figure 2:**
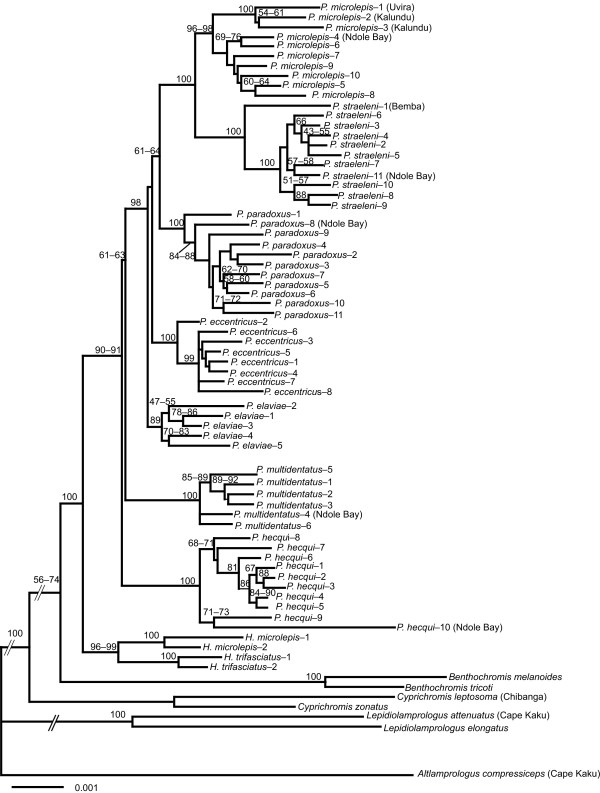
**Neighbour-joining tree based on the Nei and Li (1979) genetic distance obtained from 1582 AFLP characters**. Numbers at nodes indicate the ranges of bootstrap values using three different parameter sets for recognition site length at 10, 16, and 26 bp (values <50% not shown). Sampling localities other than the main sampling site, Kasenga, Zambia (see Figure 1) are provided in parentheses after the species names.

In total, 1582 AFLP fragments including 996 informative characters were scored for 72 individuals collected in the field (Figure 1). The neighbour-joining (NJ) analysis produced a tree (Figure 2) in which the monophyly of the tribe Perissodini, *Haplotaxodon*, and *Perissodus *was supported (BP = 100%, 96–99%, and 90–91%, respectively). The monophyly of each species was also strongly supported in this AFLP tree (BP = 89–100%). Among the *Perissodus *species, *P. hecqui *was placed as the most basal, followed by *P. multidentatus *(BP = 61–63%). The remaining five *Perissodus *species constituted a monophyletic group (BP = 98%) in which *P. straeleni *and *P. microlepis *were clustered together (BP = 100%), with *P. paradoxus *as the likely sister taxon (BP = 61–64%).

### Stomach contents analysis

**Figure 3:**
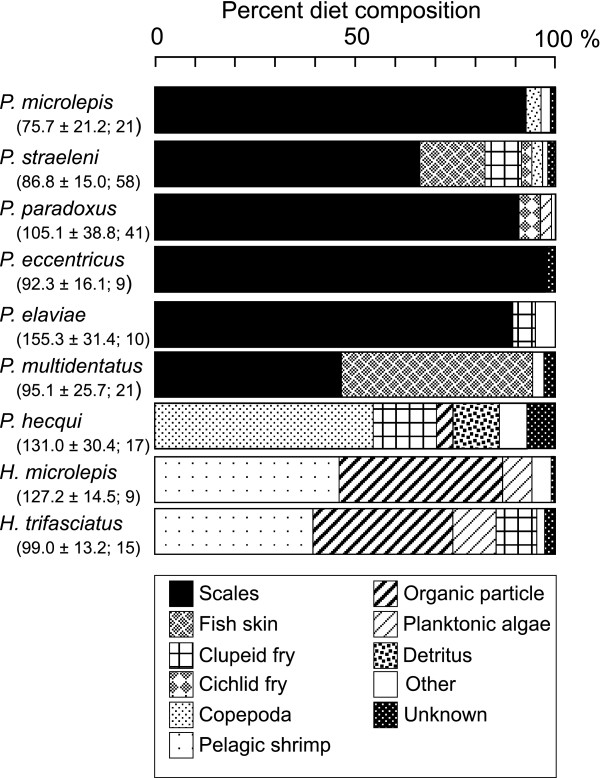
**Diet composition of Perissodini species**. The mean standard length ± standard deviation (mm) and the number of specimens examined for each species are given in parentheses. Items that comprised <2% of the diet were included in "Other".

Scales, primarily those of cichlids, were a major component in the diet of five *Perissodus *species (*P. microlepis*, *P. straeleni*, *P. paradoxus*, *P. eccentricus*, and *P. elaviae*). Except in *P. straeleni*, scales accounted for approximately 90% of the diets (Figure 3). Fry of the clupeids *Limnothrissa miodon *and *Stolothrissa tanganicae*, which are abundant in pelagic open waters [24], or those of littoral cichlids, were occasionally found in the stomach contents of these species. Fish skin (epidermal and dermal tissues) was observed in the stomach contents of *P. straeleni *(16.0%).

In contrast, the proportion of scales in the diet was lower among the basal lineages of *Perissodus *species than in the above five species. Fish skin (47.7%) and scales (46.8%; mainly of clupeids) were major diet components of *P. multidentatus*. The scales were not found alone, but always with skin. *Perissodus hecqui *appeared to be a zooplankton feeder, with calanoid copepods as its main prey (54.5%), although it also consumed clupeid fish fry (16.5%). The stomach contents of this species also contained detrital materials, including plant tissue, sponges, a few scales, pieces of fish fin, and sand grains (11.5%).

*Haplotaxodon *species fed on several prey items, with the pelagic shrimp *Mysis *sp. as the main prey (46.4% and 39.5%, respectively, in *H. microlepis *and *H. trifasciatus*), followed by aggregates of small organic particles, which included a variety of planktonic remains such as crustacean moults, plant cells, and phytoplankton. Planktonic algae, mostly blue-green algae (mainly *Microcystis *and *Anabaena*), green algae (e.g., *Gloeocystis, Coelastrum*), and diatoms, were also found in the stomach contents. Clupeid fry were also consumed by *H. trifasciatus *(10.2%).

### Habitat depths

*Perissodus microlepis *and *P. straeleni *were collected mainly from shallow rocky regions less than 70 m in depth (Table 1). *Perissodus paradoxus *was found at depths of 2 to 162 m, ranging from shallow rocky regions to deep areas. The remaining four *Perissodus *species were collected only at depths greater than 40 m. *Haplotaxodon *species were found in shallow waters.

**Table 1:** Number of times and depths of sampling for Perissodini species

	Number of individuals caught within each depth range (m)*				
Species	1–19	20–39	40–69	70–99	≥100
*Perissodus microlepis*	60 (12)	-	6(1)		
*Perissodus straeleni*	25 (9)	-	2 (1)		
*Perissodus paradoxus*	10 (4)	-	8 (4)	6 (3)	2 (1)
*Perissodus eccentricus*		-	8 (1)	4 (4)	31 (20)
*Perissodus elaviae*		-	7 (3)	1 (1)	14 (11)
*Perissodus multidentatus*		-	3 (1)	6 (3)	26 (17)
*Perissodus hecqui*		-	14 (4)	9 (6)	13 (9)
*Haplotaxodon microlepis*	20 (4)	-			
*Haplotaxodon trifasciatus*	25 (3)	-			

* The numbers of samplings conducted at each depth range are given in parentheses. In waters deeper than 40 m, gill nets were deployed for about 1 h. In shallow rocky areas less than 20 m in depth, gill nets were set using SCUBA. Sampling was not conducted at depths of 20–39 m. The anoxic region begins at depths around 120–140 m in this region.

### Morphology of the oral jaw teeth

**Figure 4:**
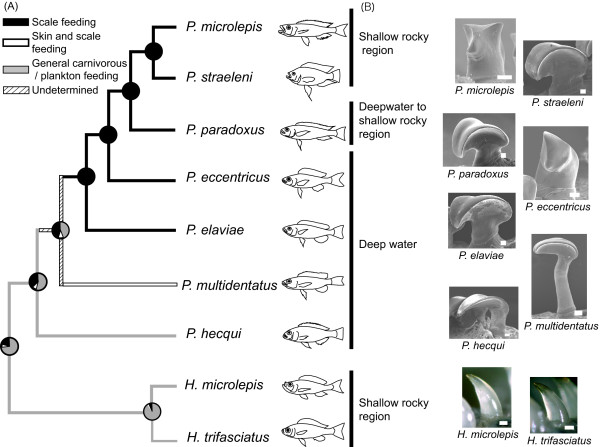
**Ancestral state reconstruction for the feeding habits (A) and the habitat depth and oral tooth shape (B) of nine species of Perissodini**. A) Ancestral state of the feeding habits estimated using the maximum parsimony method is indicated on the tree. The coloured portion in each pie diagram corresponds to the calculated probability of the reconstruction of the respective feeding habit using the maximum likelihood method. The tree used for ancestral state estimation was inferred from AFLP data. Branch lengths are not to scale in this diagram. B) The habitat depth and oral tooth shape of each species are shown to the right. The teeth were photographed with a scanning electron microscope for *Perissodus *species, and a Keyence digital microscope for *Haplotaxodon *species. Bars in the photographs indicate 0.1 mm.

**Figure 5:**
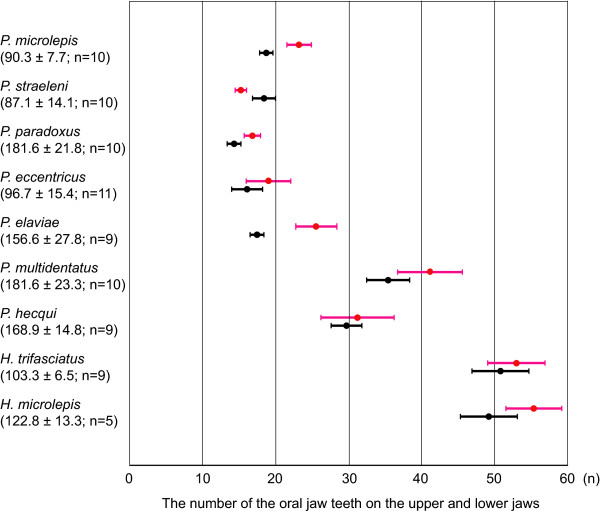
**The number of oral jaw teeth (mean ± S.D.) in Perissodini species**. The number of teeth arranged in a single row on both jaws was counted. Red bars indicate the number of teeth from the upper jaws whereas black bars indicate those from the lower jaws in respective species. The mean standard length ± standard deviation (mm) and the number of specimens examined for each species are given in parentheses.

Teeth were arranged in a single row on both the upper and lower jaws in all Perissodini species. Five *Perissodus *species, *P. microlepis*, *P. eccentricus*, *P. straeleni*, *P. paradoxus*, and *P. elaviae*, had fewer but larger teeth (see Figures 4B and 5) than did the other *Perissodus *and *Haplotaxodon *species, and these teeth were strongly recurved backwards. In *P. microlepis*, the corners of the upper side of each tooth projected vertically, forming a pair of spine-like points. In *P. eccentricus*, one side of each tooth was sharply edged with a blunt point on the tip, forming a fist-like projection. In *P. straeleni*, *P. paradoxus*, and *P. elaviae*, each tooth had a laterally widened leaf-shaped crown, forming sharp edges laterally. *P. hecqui *and *P. multidentatus *had a large number of small teeth that were also recurved backwards. The teeth of *P. multidentatus *had slim, elongated stems with right-angled crowns, whereas those of *P. hecqui *had short stems with leaf-shaped crowns. The teeth of *Haplotaxodon *species were generalised, small, conical in shape, and slightly recurved backwards.

### Ancestral reconstruction of feeding habits and morphology

The ancestral reconstruction of feeding habits based on MP and ML methods both yielded similar results. The general carnivorous/plankton feeding found in *Haplotaxodon *species and *P. hecqui *was most ancestral in Perissodini (Figure 4A). The skin-eating habit most likely evolved once in *P. multidentatus*. The specialized scale-eating habit appeared to be monophyletic in the clade containing *P. elaviae*, *P. eccentricus*, *P. paradoxus*, *P. straeleni*, and *P. microlepis*. The feeding habit prior to specialized scale eating was undetermined; ML analysis gave the probability for each character state at the ancestral node of *P. multidentatus *and the five specialized scale eaters as 0.45, 0.10 and 0.45 for general carnivorous/plankton feeding, skin eating, and scale eating respectively.

Reconstruction of tooth shapes indicated that the ancestral state was a recurved pattern in Perissodini (Figure 4B). Sharply pointed tooth patterns occurred twice, independently, in *P. microlepis *and *P. eccentricus*. Parsimony reconstruction of habitat depths unambiguously indicated that the deepwater habitat type was the most ancestral (Figure 4B) and that a transitional shift from deepwater to shallow rocky regions occurred in *Perissodus*.

## Discussion

### Phylogenetic relationships of Perissodini species

The present molecular phylogenetic study based on the AFLP method strongly supports monophyly for each species and provides important implications for the phylogenetic relationships in Perissodini (Figure 2). In the Perissodini, the genus *Haplotaxodon *was not rejected as a sister group to the genus *Perissodus *as was suggested by Poll [4]. In the genus *Perissodus*, *P. hecqui *and *P. multidentatus *appeared as basal to other *Perissodus *species, as with the morphological phylogeny of Liem & Stewart [6]. The phylogenetic relationships of the remaining five species revealed a sister relationship between *P. microlepis *and *P. straeleni*, with *P. paradoxus *likely being a sister taxon. This does not agree with the traditional classification, which was primarily based on oral tooth morphology and divides these into two groups: the "*Plecodus*" group (including *P. paradoxus*, *P. elaviae*, and *P. straeleni*), and the "*Perissodus*" group (including *P. microlepis *and *P. eccentricus*) (see Figure 4B) [4,6]. Tooth shape is an adaptive morphological trait, which could be susceptible to homoplasy through natural selection [25,26]. Thus, the traditional morphological classification for this group cannot be concluded to reflect their evolutionary relationships. Rather, recurrent evolution of similar feeding morphology appears to have occurred with specialization to scale eating, as will be discussed below.

The present AFLP phylogenetic tree agrees in part with the AFLP tree suggested by Koblmüller *et al*. [17], particularly in the sister relationship of *P. microlepis *and *P. straeleni*, and the placement of *Haplotaxodon *species as sister to *Perissodus *species. Differences in resolution and phylogenetic relationships are observed between the two studies, probably due to the limited number of AFLP characters and the lack of *P. eccentricus *in their AFLP tree [17]. Our AFLP tree obtained better resolution with over 1000 multilocus AFLP characters, although some basal nodes for *Perissodus *species were still not well resolved. This low resolution at some nodes with short branch lengths can be partly explained by rapid cladogenesis events that may have occurred at the onset of the diversification of scale-eating cichlids as previously suggested from mtDNA studies of Perissodini and other Tanganyikan tribes [17,27,28].

The mtDNA phylogeny for Perissodini was recently reported to show strong incongruence from AFLP phylogenetic trees [17]. Our phylogenetic analyses based on mitochondrial cytochrome *b *sequences support these result (see additional files 1 &2). Ancestral polymorphisms due to rapid cladogenesis events provide the most likely explanation for these discrepancies, as has been suggested for several Tanganyikan cichlids [17,29]. Furthermore, hybridisation should also be considered as a potential source for such incongruence. In our cytochrome *b *genealogy, *P. microlepis *and *P. straeleni *from the northern and southern regions clustered together, and *P. elaviae *and *P. paradoxus *shared similar haplotypes. These results suggest that past and/or recurrent hybridisation events may have occurred between these species pairs.

### Evolution of scale eating

Ancestral reconstructions of feeding habits based on our new phylogenetic framework suggest the evolution of feeding in the Perissodini from general carnivorous feeding to highly specialized scale eating (Figure 4A). *Haplotaxodon *species appear to be general carnivorous feeders that mainly collect mysid shrimp by effectively using their upwardly pointed mouth. At the basal lineage of *Perissodus*, *P. hecqui *appears to be a zooplankton feeder. This result agrees with the observation that *P. hecqui *has twice as many gill rakers as other *Perissodus *species [30]. However, the specialized recurved oral teeth of *P. hecqui*, similar to those of the other *Perissodus *species, suggest some biting function. This implies that the ancestral feeding habit of *Perissodus *species involved some carnivory. Another basal taxon, *P. multidentatus*, appears to feed on both fish skin and scales at high rates. The fish skin was always found with scales, implying that this species bites the flanks of fish, rather than simply tearing off scales. Such skin-eating may reflect an original mode of scale eating in this group.

The highly specialized scale-eating habit appears in the five remaining species, which form a monophyletic group, suggesting a single origin for specialization in this lineage (Figure 4A). Among these species, *P. straeleni *also uses other resources such as fish skins and fish fry (Figure 3). This may be an alternative feeding strategy of older, large *P. straeleni*, which may show a decreased ability to feed on scales, probably because of worn teeth [31]. In fact, scale-less non-cichlid fishes such as catfish *Chrysichthys *spp. and *Synodontis *spp. are frequent targets of skin eating by older *P. straeleni *[32]. Therefore, the most likely explanation for the variation in food habits is secondary divergence from scale eating as a consequence of supplementary feeding.

### Divergence of feeding morphology and behaviour

It has been suggested that the oral jaw tooth structure of *Perissodus *species is a unique adaptation for scale eating [6,33]. Our study further indicates that the oral tooth shapes have divergently differentiated, particularly among the specialized scale eaters. Whereas the laterally sharp-edged teeth of *P. paradoxus*, *P. elaviae *and *P. straeleni *appear to function as blades for scraping scales, the broad-based, pointed teeth of *P. microlepis *probably function as damage-resistant teeth to effectively hook and wrench off scales [31]. Such functional significance may also have promoted the convergent oral tooth structure in *P. microlepis *and *P. eccentricus*. However, whereas the teeth of these two species have similar shapes for a wrenching feeding action, the sharp edges of the teeth of *P. eccentricus *imply a somewhat different function from that of *P. microlepis*; a scraping feeding action such as that observed in *P. straeleni *may also be involved in the feeding behaviour of *P. eccentricus*.

Our study also revealed a sister relationship of the coexisting specialized scale eaters *P. microlepis *and *P. straeleni*, both of which are very common in the shallow waters of Lake Tanganyika. These species exhibit differential feeding morphologies and hunting behaviours [7]. Notably, these two species increase their hunting success by diverting the caution of the prey through diverse hunting techniques [34-36]. This situation, termed 'exploitative mutualism' [36], would play an important role in the stable coexistence of *P. microlepis *and *P. straeleni*, and may have promoted further morphological and behavioural divergence of these two species under sympatric conditions. Although nothing is known about the behaviour of deepwater scale eaters, such a relationship might also be found between the deepwater, syntopic pair of scale eaters, *P. elaviae *and *P. eccentricus*, which also differ in oral tooth shape.

### The deepwater origin of scale eating

The present study suggests a deepwater origin for scale eating. In shallow rocky habitats, scale eaters share prey species with some specialized pursuit piscivores such as *Lepidiolamprologus *spp. [37]. On the other hand, in deepwater habitats, such benthic pursuit hunters are absent, though some pelagic piscivorous cichlids such as *Bathybates *spp. and general carnivorous cichlids such as *Telotrematocara macrostoma *are common [11]. Thus, the feeding niche, which is dominated by the specialized pursuit hunters in the shallow habitat, seems to be vacant in the deepwater habitat. It can therefore be speculated that such a niche may have been exploited by the ancestor of deepwater scale-eating cichlids. Their large recurved oral jaw tooth shape, which differs from the conical tooth shape of true piscivorous species, may also have been suited for biting off small portions from their prey's flank. Such morphological constraints might also have led this group to further specialization of the scale eating habit.

## Conclusion

Although scale-eating cichlids also inhabit other younger African lakes, i.e., Lake Victoria and Lake Malawi, the number of species and the degree of specialization for scale eating are greatest in the *Perissodus *species of Lake Tanganyika [1,11]. Our study revealed the phylogenetic relationships of the Perissodini, and based on the resultant tree, for the first time, proposed a comprehensive evolutionary sequence for the specialization of scale-eating habits. The mtDNA phylogenetic tree suggests that the diversification of *Perissodus *species occurred roughly in the late Neogene (1.7–7 Mya), implying that these species may have experienced the dramatic geological events of the lake, including the lake level changes that occurred 2.5–3 Mya, as discussed for Bathybatini [38]. The remarkable diversity of *Perissodus *could be attributed to the complex geological history of Lake Tanganyika and complex interspecific relationships among fishes in the lake.

## Methods

### Specimens

Specimens of all nine described species from the tribe Perissodini (two *Haplotaxodon *and seven *Perissodus*), and representatives of lineages nested close to the Perissodini in the analysis of Salzburger *et al*. [14] (two cyprichromine species, two benthochromines, and three lamprologines), were collected at Kasenga, Zambia, and from several other sites in Lake Tanganyika (Figure 1). Specimens were collected using gill nets, anaesthetised by storing in an icebox, and preserved in 99% ethanol. Specimens for the analysis of stomach contents were injected with 37% formaldehyde solution into the stomach and were subsequently preserved in 10% formaldehyde solution. Specimens for the observation of jaw morphology were also preserved in 10% formaldehyde solution. These collection procedures were approved under the guidelines for animal experiments enacted by the Ministry of Education, Culture, Sports, Science and Technology, Japan (MEXT).

The habitat depths of all Perissodini species were estimated from collection records conducted at various water depths near Kasenga, Zambia. These samples were collected using gill nets (see Table 1).

### Amplified fragment length polymorphism (AFLP) analysis

The AFLP analysis followed a protocol modified from Vos *et al*. [39]. The AFLP Plant Mapping Kit protocol (Applied Biosystems, Foster City, CA, USA) was used. DNA digestion was performed using *Eco*RI (20 units; New England Biolaboratories, Beverly, MA, USA), and *Mse*I (5 units) at 37°C for 5 h in a thermal cycler. At the end of 5 h, a ligation reaction was performed with a restriction mixture containing each *Eco*RI and *Mse*I adapter at 16°C overnight. Pre-selective amplification with one selective base on each primer (*Eco*RI-A and *Mse*I-C) and 11 different selective amplifications was performed using the following combinations of primers with two additional bases (CT-TT, CG-TT, CA-TT, CG-TG, GG-TC, CT-TA, CA-TA, GG-AC, CT-AC, CA-AC, AG-AC). PCR was performed on a PC808 thermal cycler (ASTEC, Fukuoka, Japan). The DNA concentration was checked prior to restriction reactions. Fragments were electrophoresed on an ABI Prism 310 Genetic Analyzer (Applied Biosystems) with internal size standards (GS 500 ROX; Applied Biosystems). Signal detection was carried out using GeneScan ver. 3.1 (Applied Biosystems). The fluorescence threshold was set to 50 r.f.u. and the correct fit of size standards was checked for all electropherograms. Scoring to presence/absence was conducted between 50 and 499 bases using Genotyper ver. 2.5 (Applied Biosystems). Peaks with values <0.4 were considered the same.

Nei and Li [40] genetic distances were calculated with the site (nucleotide) length set at 16 using the program Restdist in PHYLIP ver. 3.65 [41]. Trees were constructed using the NJ algorithm [42] implemented in Neighbour in PHYLIP. Alternative restriction site length parameters of 10 and 26 for the distance program were also used to examine the robustness of the distance model. To assess the robustness of the NJ tree, 1000 bootstrap replications were conducted with each site length parameter using Seqboot and Neighbour in PHYLIP.

### Stomach contents analysis

Stomach fullness was assessed under a light microscope according to Hynes' [43] point method with minor modification, i.e., 4 points for 1/4 fullness, 8 points for 1/2 fullness, 16 points for complete (1×) fullness, and 32 points for twice (2×) fullness. After each food item in the stomach was identified, its volume relative to the fullness points was judged. Points were allotted to each food item according to the relative volume. The percent contribution of each food item in each species was calculated by pooling the total points for each food item and dividing by the sum of stomach fullness points.

### Observation of oral jaw tooth morphology

The oral jaws were removed from one specimen for each *Perissodus *and *Haplotaxodon *species, cleaned with water and dehydrated in 70% ethanol. The oral jaw teeth were then observed and photographed using a scanning electron microscope (SEM; JSM5800, JEOL, Tokyo, Japan) for *Perissodus *species, and a digital microscope (VHX-100, Keyence, Osaka, Japan) for *Haplotaxodon *species. The teeth were coated with gold prior to SEM photographing. The numbers of teeth on both jaws were also enumerated under a binocular microscope. Full adults with relatively few missing teeth were used to minimise the effects of natural replacement or wearing out of the teeth.

### Ancestral state reconstruction

Ancestral reconstruction of feeding habits, oral tooth morphology and habitat depths were undertaken using the NJ tree from the AFLP dataset. For reconstruction of feeding habits, oral jaw tooth morphologies, and habitat depths, maximum parsimony ancestral reconstruction was performed using Mesquite [44]. Additionally, maximum-likelihood ancestral reconstruction was performed for reconstruction of ancestral states of feeding habits, using Mesquite, based on a Markov k-state one-parameter model [44].

## Competing interests 

The author(s) declares that there are no competing interests.

## Authors' contributions

RT collected the data, performed the analyses, and wrote the first draft of the manuscript. KW conducted and supervised the molecular work and analyses, and supervised the writing of the draft manuscript. MN established the study design and helped write the final draft of the manuscript. MH conducted the study design and coordination, and supervised the ecological analyses and writing of the draft manuscript. KW, MN, and MH performed the collection of samples. All authors read and approved the final manuscript.

## Supplementary Material

Additional file 1 Maximum likelihood tree based on mitochondrial cytochrome *b *gene sequences. Numbers at nodes correspond to bootstrap probabilities (values ≤50% not shown) on the left and Bayesian posterior probabilities on the right. The 63 specimens used for this study correspond to those for the AFLP analyses although two *Perissodus *specimens (*P. eccentricus*-1 and *P. elaviae*-5) were omitted from the mtDNA tree because of failure in mtDNA PCR reactions. Sampling localities other than the main sampling site, Kasenga, Zambia (see Figure 1), and the accession numbers are provided in parentheses after the species names. Click here for file

Additional file 2 Materials & Methods, and Results for mtDNA cytochrome *b *gene analysis. The data provided give the descriptions of Materials & Methods, and results for mtDNA phylogenetic analysis. Click here for file
