# Supplementary material for: Evolution of feeding specialization in Tanganyikan scale-eating cichlids: a molecular phylogenetic approach
Source: BMC Evol Biol. 2007 Oct 18;7:195. doi: 10.1186/1471-2148-7-195 (PMC2212659; doi:10.1186/1471-2148-7-195)
Supplement: Additional file 1 — Maximum likelihood tree based on mitochondrial cytochrome b gene sequences. Numbers at nodes correspond to bootstrap probabilities (values ≤50% not shown) on the left and Bayesian posterior probabilities on the right. The 63 specimens used for this study correspond to those for the AFLP analyses although two Perissodus specimens (P. eccentricus-1 and P. elaviae-5) were omitted from the mtDNA tree because of failure in mtDNA PCR reactions. Sampling localities other than the main sampling site, Kasenga, Zambia (see Figure 1), and the accession numbers are provided in parentheses after the species names. [file 1471-2148-7-195-S1.pdf]

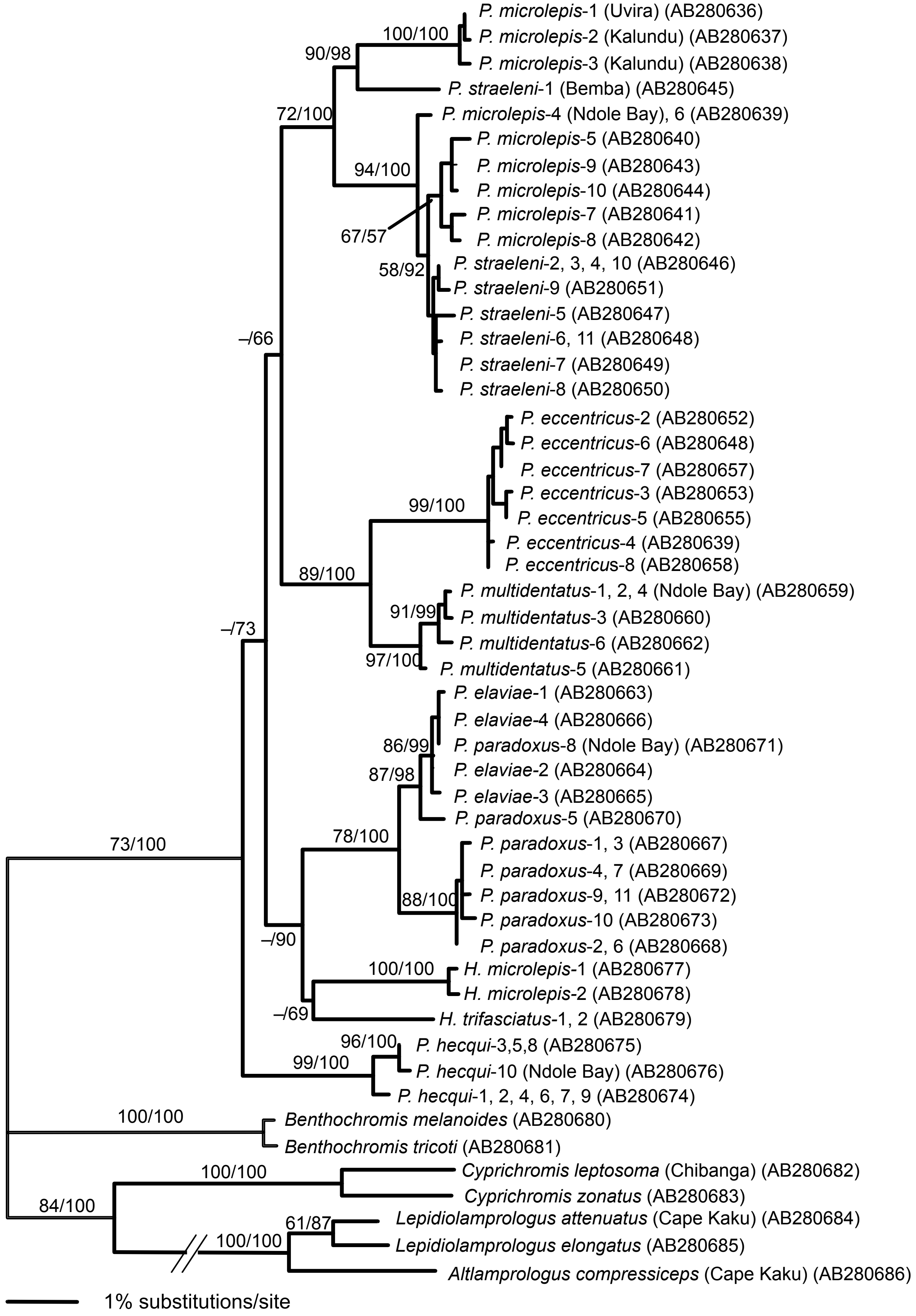

Evolution of feeding specialisation in Tanganyikan scale-eating cichlids: a molecular phylogenetic approach

By R. Takahashi, K. Watanabe, M. Nishida, and M. Hori

Additional file 1–Supplementary figure 1. Maximum likelihood tree based on mitochondrial cytochrome *b* gene sequences.
