# Supplementary material for: Evolution of feeding specialization in Tanganyikan scale-eating cichlids: a molecular phylogenetic approach
Source: BMC Evol Biol. 2007 Oct 18;7:195. doi: 10.1186/1471-2148-7-195 (PMC2212659; doi:10.1186/1471-2148-7-195)
Supplement: Additional file 2 — Materials & Methods, and Results for mtDNA cytochrome b gene analysis. The data provided give the descriptions of Materials & Methods, and results for mtDNA phylogenetic analysis. [file 1471-2148-7-195-S2.pdf]

Evolution of feeding specialisation in Tanganyikan scale-eating cichlids: a molecular phylogenetic approach

By R. Takahashi, K. Watanabe, M. Nishida, and M. Hori

Additional file 2 – Results and Methods for mtDNA cytochrome *b* gene analysis

Note: The phylogenetic tree of mtDNA analysis is given in Additional File 1.

Results of mtDNA phylogeny

The inferred maximum likelihood (ML) and Bayesian trees were mostly identical. Although the resolution at the basal nodes of Perissodini was generally low, the monophyly of Perissodini (BP = 73% in ML; PP = 100% in BI), the sister relationship of *P. multidentatus* and *P. eccentricus* (89%; 100%) and the monophyly of the *P. microlepis*/*P. straeleni* complex (72%; 100%) and the *P. elaviae*/*P. paradoxus* complex (78%; 100%) were well supported. Note that the latter four *Perissodus* species did not constitute a monophyletic group: *P. microlepis* and *P. straeleni*, from the northern and southern regions, clustered together, whereas *P. elaviae* and *P. paradoxus* shared similar haplotypes. *Haplotaxodon* species were included in a subclade of the *Perissodus* cluster, although the monophyly of *Perissodus* was not rejected by the Shimodaira-Hasegawa (S-H) [1] test (Δ –ln L = 1.686, P = 0.315). The monophyly of each species in the Perissodini was also rejected by the S-H test (Δ –ln L = 61.957, P = 0.001).

The evolutionary distance (TrN+I+G distance) for basal divergence in the *Perissodus* was around 5–7% substitutions/site. Applying previously suggested fish molecular clocks of 1–3% pair-wise distance/Myr [2-4], the divergence time scale was roughly estimated at 1.7–7 Myr ago.

Methods for mtDNA amplification, sequencing and phylogenetic analyses

Partial sequences of the mitochondrial cytochrome *b* gene (1133 bp) were used to construct the mtDNA phylogeny of the Perissodini. Polymerase chain reaction (PCR)

amplification was carried out using the primer pair L14724 (5'-TGACTTGAARAACCAYCGYYG-3') and H15915 (5'-ACCTCCGATCTYCGGATTACAAGAC-3') . The PCR conditions consisted of 30 cycles of denaturation (94°C, 15 s), annealing (47°C, 15 s) and extension (72°C, 30 s) on a PC808 thermal cycler (ASTEC, Fukuoka, Japan). After purifying the PCR products by treatment with ExoSAP-It (USB Corp., Cleveland, OH, USA), they were sequenced on an automated DNA sequencer (ABI Prism GA310; Applied Biosystems, Foster City, CA, USA) using amplification primers and the BigDye Terminator Cycle Sequencing FS Ready Reaction Kit ver. 1.1 (Applied Biosystems). The nucleotide sequences were deposited in DDBJ/EMBL/GenBank (Accession numbers: AB280636–AB280686).

The DNA sequences obtained were edited using the multiple-sequence editor DNASIS (Hitachi, Tokyo, Japan) and aligned by hand. Maximum likelihood (ML) and Bayesian inference (BI) methods were applied to infer the mtDNA phylogenetic tree. The TrN+I+G model [5] model for 43 haplotypes detected from 62 perissodine specimens in the ML analysis were selected by hierarchical likelihood ratio tests (hLRTs) and the Akaike information criterion (AIC) using Modeltest ver.3.07 with the following parameters: base frequencies of A = 0.229, C = 0.350, G = 0.142, and T = 0.280; proportion of invariant bases = 0.628; gamma shape = 1.024; and a substitution matrix of transversion 1 of A → G 29.823 and C → T 16.502. The ML tree was estimated using PAUP* ver.4.0b10 with 200 bootstrap replications to evaluate the robustness of the internal branches.

References

1. Shimodaira H, Hasegawa M: **Multiple comparisons of log-likelihoods with applications to phylogenetic inference.** *Mol Biol Evol* 1999, **16**:1114-1116.
2. Rocha-Olivares A, Rosenblatt RH, Vetter RD: **Molecular evolution, systematics, and zoogeography of the rockfish subgenus *Sebastomus* (Sebastes, Scorpaenidae) based on mitochondrial cytochrome b and control region sequences.** *Mol Phylogenet Evol* 1999, **11**:441-458.
3. Zardoya R, Doadrio I: **Molecular evidence on the evolutionary and**

biogeographical patterns of European cyprinids. *J Mol Evol* 1999, **49**(2):227-237.

4. Watanabe K, Mori S, Nishida M: **Genetic relationships and origin of two geographic groups of the freshwater threespine stickleback, 'hariyo'.** *Zool Sci* 2003, **20**(2):265-274.
5. Tamura K, Nei M: **Estimation of the number of nucleotide substitutions in the control region of mitochondrial DNA in humans and chimpanzees.** *Mol Biol Evol* 1993, **10**:512-526.
